# Conditioned medium from umbilical cord mesenchymal stem cells induces migration and angiogenesis

**DOI:** 10.3892/mmr.2015.3409

**Published:** 2015-03-04

**Authors:** CHONGYANG SHEN, PUCHANG LIE, TIANYU MIAO, MEIXING YU, QIAO LU, TING FENG, JINRONG LI, TINGTING ZU, XIAOHUAN LIU, HONG LI

**Affiliations:** 1 Key Laboratory of Obstetric, Gynecologic, Pediatric Diseases and Birth Defects of the Ministry of Education, West China Second University Hospital, Sichuan University, Chengdu, Sichuan 610041, P.R. China; 2 Key Laboratory of Regeneratative Biology, Guangzhou Institutes of Biomedicine and Health, Chinese Academy of Sciences, Guangzhou, Guangdong 510530, P.R. China; 3 West China School of Medicine, Sichuan University, Sichuan University, Chengdu, Sichuan 610041, P.R. China; 4 Department of Pediatrics, West China Second University Hospital, Sichuan University, Chengdu, Sichuan 610041, P.R. China

**Keywords:** umbilical cord mesenchymal stem cells, paracrine, angiogenesis, migration

## Abstract

Umbilical cord mesenchymal stem cells (UC-MSCs) have been suggested as a candidate for various clinical applications, however, major limitations include the lack of organ-specific accumulation and low survival rates of transplanted cells. In the present study, it was hypothesized that the paracrine effects of UC-MSCs may enhance stem cell-based tissue repair and regeneration by promoting the specific homing of stem/progenitor cells and the overall ability to drive them to the damaged area. UC-MSCs-derived conditioned medium (UC-CM) was analyzed using liquid chip and ELISA techniques. *In vitro* tube formation assays of human umbilical vein endothelial cells (HUVECs) and UC-MSCs were then performed to assess the angiogenic properties of UC-CM. Subsequently, UC-MSCs, HUVECs and fibroblasts were labeled with PKH26 for an *in vivo* cell migration assay. The expression levels of C-X-C chemokine receptor 4 (CXCR4), C-C chemokine receptor 2 (CCR2) and c-met were determined in the UC-MSCs, HUVECs and fibroblasts using reverse transcription-quantitative polymerase chain reaction and flow cytometry. UC-CM was incubated with or without antibodies, and the contribution of stromal cell-derived factor 1 (SDF-1), monocyte chemotactic protein 1 (MCP-1) and hepatocyte growth factor (HGF) on the migration of cells was investigated *in vitro*. The results demonstrated that UC-MSCs secreted different cytokines and chemokines, including increased quantities of SDF-1, MCP-1 and HGF, in addition to the angiogenic factors, vascular cell adhesion protein-1, interleukin-8, insulin-like growth factor-1 and vascular endothelial growth factor. The total lengths of the tubes were significantly increased in the UC-MSCs and HUVECs incubated in UC-CM compared with those incubated in Dulbecco’s modified Eagle’s medium. *In vivo* cell migration assays demonstrated that UC-CM was a chemotactic stimulus for the UC-MSCs and HUVECs. *In vitro* Matrigel migration and scratch healing assays demonstrated that UC-CM increased the migration of CXCR4-postive or/and CCR2-positive cells in a dose-dependent manner. In addition, different molecules were screened under antibody-based blocking migration conditions. The data revealed that the SDF-1/CXCR4 and MCP-1/CCR2 axes were involved in the chemoattractive activity of UC-CM and suggested that the effective paracrine factor of UC-CM is a large complex rather than a single factor. The results of the present study supported the hypothesis that UC-MSCs release soluble factors, which may extend the therapeutic applicability of stem cells.

## Introduction

Umbilical cord mesenchymal stem cells (UC-MSCs) can be easily isolated from the umbilical cord and expanded *in vitro*, and are widely used in stem cells therapy (1,2). However, the mechanisms behind their therapeutic benefits remain to be elucidated. Initially, the promising effects of UC-MSCs were based on their multipotent differentiation ability or paracrine effects (3), however, the retention of MSCs is poor, and their low survival rates in injured tissues reduces their therapeutic effects (4). This suggests that the paracrine effect of MSCs may be important in the replacement of damaged cells (5–9). Therefore, it is essential to identify strategies, which can enhance the effectiveness of MSC-based therapies, which requires elucidation of the molecular pathways responsible for MSC-mediated tissue repair.

The mechanisms by which the paracrine effects of MSCs contribute to their therapeutic effects are at present, unclear. It has been suggested that paracrine factors may mediate regeneration via the activation and recruitment of resident/circulating stem cells and progenitor cells to the site of injury, where they collaborate to heal damaged tissues (10,11). A number of studies have demonstrated that stromal cell-derived factor-1 (SDF-1) is critical for stem/progenitor cell migration. For example, the SDF-1/C-X-C chemokine receptor 4 (CXCR4) axis has been reported to promote the recruitment of progenitor cells and CXCR4-positive cells to lesions in the heart and brain (12,13). Hepatocyte growth factor (HGF) is a chemokine, which exhibits chemoattractive properties via interactions with its receptor c-met, which can induce the proliferation and migration of epithelial cells and MSCs (14,15). Monocyte chemoattractant protein-1 (MCP-1) is a potent chemoattractant, which recruits MSCs and induces the proliferation of fibroblasts (16,17). However, the paracrine actions of UC-MSCs remain to be elucidated. In particular, the involvement of the SDF-1/CXCR4, MCP-1/C-C chemokine receptor 2 (CCR2) and HGF/c-met axes in the therapeutic effects of MSCs as chemoattractants has not been investigated. Several studies have demonstrated that circulating MSCs are attracted to sites of damage, where they undergo tissue-specific differentiation (18). Progenitor cells possess the capacity to differentiate into endothelial cells and are considered to be relevant in revascularization (19). Fibroblasts are the predominant type of stromal cell in tissues, and they contribute to scar healing in injured tissues (20,21). Therefore, the present study hypothesized that an increase in the level of paracrine factors secreted from UC-MSCs in injured tissue may promote the recruitment of circulating mesenchymal stem and progenitor cells to the injured tissue.

## Materials and methods

### Isolation and culture of cells

The present study was approved by the ethics committee of the West China Second University Hospital (Chengdu, China). Umbilical cords were collected from patients who had undergone full-term cesarean-section (n=5, 26–31 years old) with their written informed consent at the West China Second University Hospital. UC-MSCs were isolated, as described previously, with certain modifications (1). Briefly, the umbilical cords were sterilized by immersion in 1% povidone-iodine (Sichuan Kelun Pharmaceutical Co., Ltd., Chengdu, China) for 2 min and were rinsed three times with phosphate-buffered saline (PBS; GE Healthcare Life Sciences, Logan, UT, USA). Wharton’s jelly was cut into 30–40 small sections (2–5 mm) and was cultured in 5% CO_2_ at 37°C in Dulbecco’s modified Eagle’s medium (DMEM; Basalmedia Technologies Co., Ltd., Shanghai, China) supplemented with 10% fetal bovine serum (FBS; GE Healthcare Life Sciences), 100 U/ml penicillin G and 100 mg/ml streptomycin (Invitrogen Life Technologies, Carlsbad, CA, USA). At 75% confluence, the cells were passaged with 0.25% trypsin (GE Healthcare Sciences). The medium was replaced every 3 days. To isolate the HUVECs, the cord vein was flushed with PBS and digested with 100 mg/ml collagenase (Sigma-Aldrich, St. Louis, MO, USA) at 37°C for 15 min. The cells, which were isolated from the cord veins, were cultured in endothelial growth media-2 (Lonza Group Ltd., Basel, Switzerland) with 100 U/ml penicillin G and 100 mg/ml streptomycin, at 37°C and 5% CO_2_. The medium was replaced every 3 days. The fibroblasts used in the present study were obtained from Dr J Chen (Cobaxer Biotechnology Co., Ltd., Chengdu, China). The cells were cultured in high-glucose DMEM (Basalmedia Technologies Co., Ltd.) supplemented with 10% FBS, 100 U/ml penicillin G, 100 mg/ml streptomycin and 3 ng/ml basic fibroblast growth factor (FGF; Invitrogen Life Technologies) and were maintained at 37°C with 5% CO_2_. As the positive control of expression of the CXCR4 and CCR2 genes, CD3-activated peripheral blood mononuclear cells were kindly provided by Dr J Chen (Cobaxer Biotechnology). 1×10^7^ PBMCs were isolated and cultured in RPMI 1640 medium (Basalmedia Technologies Co., Ltd.) containing 10% FBS and stimulated by 100 ng/ml mouse anti-human CD3 monoclonal antibody (cat. no. 317315, BioLegend, Inc., San Diego, CA, USA) and 100 IU/ml IL-2 (Invitrogen Life Technologies). Fresh RPMI 1640 medium was added every 2 days.

### Preparation of UC-CM

In order to obtain the UC-CM, UC-MSCs at passage four were seeded at a density of 10,000 cells/cm^2^. At 80% confluence, the cells were washed three times with PBS and the media were replaced with serum-free DMEM. After 72 h, the media were centrifuged (Eppendorf, Hauppauge, NY, USA) at 300 × g for 5 min, filtered through a 0.22 *μ*m filter (Pall Corporation, Port Washington, NY, USA) and were then stored at −70°C until use. For the *in vivo* assays the conditioned media were concentrated 10-fold using an ultrafiltration membrane with a molecular weight cut-off of 3 kDa (Pall Corporation, Port Washington, NY, USA).

### Growth factor assays

To analyze the types and levels of the accumulated factors and cytokines released by the UC-MSCs, the conditioned media were analyzed using ELISA and liquid chip assays. The levels of insulin-like growth factor (IGF)-1, HGF, SDF-1, interleukin (IL)-8, brain-derived neurotrophic factor (BDNF), vascular cell adhesion protein (VCAM)-1 and transforming growth factor (TGF)-β in UC-CM were measured using ELISA kits (Human IGF-1 ELISA, human BDNF ELISA, human TGF-β ELISA, RayBiotech, Inc., Norcross, GA, USA; and human CXCL12/SDF-1α quantikine ELISA kit, human HGF quantikine ELISA kit, human VCAM-1 quantikine ELISA kit, R&D Systems, Inc., Minneapolis, MN, USA). Briefly, 200 *μ*l UC-CM or serum-free DMEM was added to 96-well plates coated with monoclonal antibodies specific to the factor of interest, and the plates were incubated at 4°C for 3 h. Subsequent to washing with PBS, the antibodies were added to each well, incubated for 1 h at 4°C, and washed with wash buffer (PBS with 0.05% Tween-20). Substrate solution (3,3′,5,5′-tetramethylbenzidine) was then added, followed by stop solution (0.16 M sulfuric acid) after 45 min. The concentrations of cytokines and growth factors were calculated by measuring the absorbance at 450 nm using a microplate reader (Multiskan; Thermo Fisher Scientific, Waltham, MA, USA). The levels of stem cell factor, epidermal growth factor, FGF-2, TGF-α, IL-10, platelet-derived growth factor-BB (PDGF-BB), interferon-inducible protein-10, MCP-1 and vascular endothelial growth factor (VEGF) were detected using liquid chip kits (Human Cytokine Magnetic kit; EMD Millipore, Billerica, MA, USA) and the BeadXpress Reader system (Illumina, Inc., San Diego, CA, USA), according to the manufacturer’s instructions.

### Tube formation assay

Tube formation was assessed, as described previously (22) with certain modifications using an *in vitro* angiogenesis assay kit (EMD Millipore). The HUVECs and UC-MSCs (3×10^5^ cells/well) were incubated in 24-well plates coated with Matrigel (BD Biosciences, Franklin Lakes, NJ, USA) for 12 h in serum-free DMEM or UC-CM. Image J version 1.45S software (National Institutes of Health, Bethseda, MA, USA) was then used to measure the total tube length on the captured images (magnification, ×40) by microscopy (CKX31; Olympus Corporation, Tokyo, Japan).

### In vivo migration assay

To investigate the chemotactic properties of UC-CM, *in vivo* migration models were constructed, using stem cells and other progenitor cells as targets to identify UC-CM-induced cell migration. All animal experiments were performed in accordance with the ethics committee of the West China Second University Hospital. A total of 60 male 10-week-old C57BL/6 mice (weighing 25–30 mg; Experimental Animal Center of Sichuan University, Chengdu, China) were maintained in an artificially ventilated environment (temperature, 20–26°C; light intensity, 180–300 lux), and were fed palatable and uncontaminated diets *ad libitum*. The mice were anesthetized with 10% chloral hydrate (Tokyo Chemical Industry Co., Ltd., Tokyo, Japan) (0.1 ml/10 g). A total of 300 *μ*l ice-cold growth factor-reduced Matrigel was combined with 200 *μ*l concentrated UC-CM or DMEM as a control, which was subcutaneously injected into the left side of each mouse’s back using an insulin syringe fitted with a 23G needle (BD Biosciences) (n= 5). The injections were performed slowly, allowing the Matrigel to polymerize and form a jelly-like implant under the skin. Prior to cell implantation, the cultured fibroblasts, HUVECs and UC-MSCs were detached using 0.25% trypsin, and stained with PKH26 (Sigma-Aldrich). The fibroblasts, HUVECs and UC-MSCs were diluted (1×10^6^ cells/100 *μ*l saline) 2 h following Matrigel implantation, and were then subcutaneously injected into the 1 cm area surrounding the Matrigel implants.

### Immunohistochemistry

To quantify the cell migration into the Matrigel implants, the mice were sacrificed by isoflurane inhalation (Sichuan Kelun Pharmaceutical Co., Ltd.) 8 and 16 days subsequent to injection. The whole Matrigel was then isolated and fixed in 4% paraformaldehyde (Sigma-Aldrich) overnight, followed by 30% sucrose/phosphate buffer (Sigma-Aldrich) for 24 h, prior to being embedded in optimum cutting temperature medium (Sakura Finetek Europe B.V., Alphen aan den Rijn, The Netherlands). The frozen Matrigel was cut into 10 mm sections using a cryostat (LEICA CM3050S; Leica Microsystems Inc., Buffalo Grove, IL, USA), and directly photographed (magnification, ×40) by fluorescence microscopy (DMI3000 B; Leica Microsystems, Inc.). The total numbers of migrated cells were then counted in three randomly selected fields.

### Flow cytometry

Fibroblasts, HUVECs and UC-MSCs were harvested using 0.25% trypsin, washed and resuspended in PBS containing 1% bovine serum albumin (BSA; Sigma-Aldrich). Cells were stained with PerCP/Cy5.5-conjugated mouse anti-human CCR2 (cat. no. 335303; BioLegend, Inc.), PE-conjugated mouse anti-human CXCR4 (cat. no. 306505; BioLegend, Inc.) and fluorescein isothiocyanate-labeled mouse anti-human c-met (cat. no. 11-8858; eBioscience, Inc., San Diego, CA, USA), according to the manufacturer’s instructions. The cells were then analyzed using flow cytometry (Gallios; Beckman Coulter, Brea, CA, USA) and FlowJo software, version 7.6 (FlowJo, LLC, Ashland, OR, USA).

### RNA extraction and reverse transcription-quantitative polymerase chain reaction (RT-qPCR)

Total RNA was isolated using an RNeasy mini kit (Qiagen, Shanghai, China), according to the manufacturer’s instructions. The RNA was incubated with DNase I (Invitrogen Life Technologies) in order to eliminate any genomic DNA contamination. The total RNA was then reverse transcribed using the SuperScript III First-Strand Synthesis kit (Invitrogen Life Technologies). cDNA was analyzed by PCR using 20 ng cDNA in a 50 *μ*l reaction volume containing primers and Ex-Taq DNA polymerase (Takara Biotechnology Co., Ltd., Dalian, China). The PCR conditions included 32 cycles of 94°C for 60 sec, 58°C for 60 sec and 72°C for 90 sec. GAPDH was used as the housekeeping control gene. The following primers (Invitrogen Life Technologies) were used (14,23): CXCR4, forward ATGGAGGGGATCAGTATATACAC and reverse TGGAGTGTGCTATGTTGGCGTCT; c-met, forward GGGTCGCTTCATGCAGGTTGTGGT and reverse ATGGTCAGCCTTGTCCCTCCTTCA; CCR2, forward CCAACGAGAGCGGTGAAGAAGTC and reverse TCCGCCAAAATAACCGATGTGAT; GAPDH, forward GCCAAGGTCATCCATGACAACTTTGG and reverse GCCTGCTTCACCACCTTCTTGATGTC.

### Chemoinvasion assay

A chemoinvasion assay was performed to evaluate the ability of cells to cross a Matrigel membrane. The upper chambers, with 8 mm pores, were coated with 50 *μ*l Matrigel diluted 1:10 (v:v) in DMEM and were incubated at 37°C for 4 h. The lower chambers contained either DMEM supplemented with 1% BSA as a control or UC-CM. For specific factor blocking assays, 20 *μ*g/ml each of the monoclonal mouse anti-human anti-SDF-1 (cat. no. MAB350; R&D Systems, Inc.), anti-MCP-1 (cat. no. 16-7096; eBioscience, Inc.) and anti-HGF (eBioscience, Inc.) antibodies were added to the lower chambers. The fibroblasts, HUVECs and UC-MSCs were prepared in DMEM supplemented with 1% BSA, and 5×10^4^ cells in 0.5 ml suspension were added to each upper chamber. Each experiment was performed in triplicate. The chambers were placed in a 24-well plate and were incubated at 37°C, with 5% CO_2_ for 24 h. The cells, which had not crossed the membrane were removed with a wet cotton bud. The undersides of the filters were then fixed in methanol (Sigma-Aldrich) for 10 min and stained with 0.1% crystal violent (Sigma-Aldrich), and images of the cells, which had invaded to the underside of the insert were captured. Three random fields were selected (magnification, ×40) by microscopy (CKX31; Olympus Corporation) and counted.

### Scratch healing assay

A 24-well plate was coated with 8 mg/cm^2^ collagen I (Sigma-Aldrich) for 2 h at 37°C, excess fluid was removed from the coated surface and the plate was dried overnight. Following this, fibroblasts, HUVECs and UC-MSCs were incubated in pre-coated plates (2×10^5^ cells/well) and individually maintained at 37°C with 5% CO_2_ for 24 h in serum-free DMEM or UC-CM. A yellow pipette tip was then used to scratch the confluent monolayers. The media were replaced with fresh medium and the scratch was analyzed after 6 h using ImageJ software.

### Statistical analyses

Data are expressed as the mean ± standard error of the mean. Statistical comparisons were performed using Student’s t-test. P<0.05 was considered to indicate a statistically significant difference. One-way analysis of variance with Bonferroni’s post hoc test was used to compare the migration of cells *in vivo*. ^*^P<0.05, ^**^P<0.01 and ^***^P<0.001 vs. control group.

## Results

### Cytokine release from the UC-MSCs

To determine which migratory and angiogenic factors were secreted by the UC-MSCs, the cytokine content of UC-CM was measured using ELISA and liquid chip assays (Table I). Compared with the fibroblasts, the UC-MSCs expressed markedly increased levels of chemoattractant factors, including SDF-1, MCP-1, TGF-β, PDGF-BB, VEGF, VCAM-1 and MCP-1. In particular, the levels of SDF-1, MCP-1 and HGF were higher in the UC-MSCs compared with the fibroblasts. In addition, UC-CM contained significantly increased levels of several angiogenic factors, including IL-8, IGF-1 and VEGF. However, IL-10, an immunoregulatory factor, was not detected in the UC-CM.

### UC-CM enhances angiogenesis in vitro

**Figure 1:**
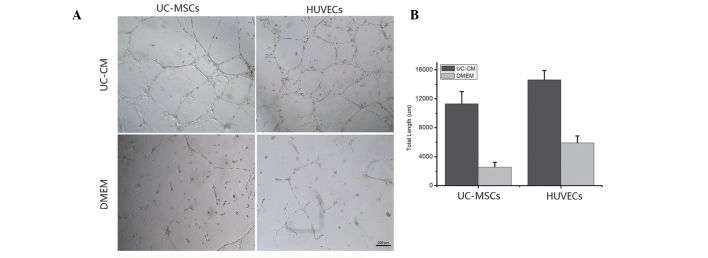
Matrigel tube formation assay. UC-MSCs and HUVECs were cultured for 12 h in 24-well plates coated with a semi-solid matrigel. The cells were cultured in (A) UC-CM or DMEM (as a control). (B) Total tube lengths formed in the assay were measured. ^*^P<0.05, vs. DMEM group, according to Student’s t-test. Scale bar=200 *μ*m. Data are presented as the mean ± standard error of the mean. UC-MSCs, umbilical cord mesenchymal stem cells; HUVECs, human umbilical vein endothelial cells; UC-CM, UC-MSC conditioned medium; DMEM, Dulbecco’s modified Eagle’s medium.

To investigate the angiogenic effects of UC-CM, a tube formation assay was performed to form vascular networks. The UC-MSC and HUVEC tube formations were then quantified by counting the total length of the formed networks (Fig. 1). The UC-MSCs and HUVECs grown in DMEM only (control) did not form complex tubular structures, whereas the cells cultured in UC-CM formed tubules and tubular rings. The total length of the tubes was significantly increased in the UC-MSCs and HUVECs incubated with UC-CM compared with those incubated with DMEM. The UC-CM stimulated the formation of HUVEC tubular networks as early as 4 h following seeding onto the matrix, and the structures were maintained for a minimum of 36 h. The UC-CM was less efficient at stimulating the growth of UC-MSC tubular structures, which were visible after 10 h and lasted for 24 h.

### UC-CM increases the in vivo migration of transplanted cells

**Figure 2:**
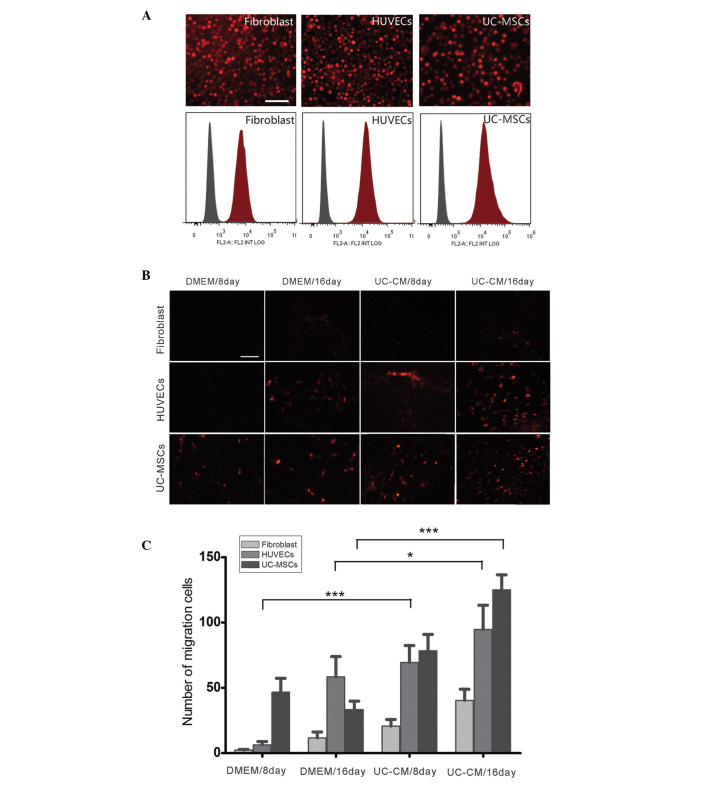
*In vivo* migration assay. (A) Staining of fibroblasts, HUVECs and UC-MSCs with PKH26. Labeling was quantified using flow cytometry. High levels of red fluorescence were observed in ~95% of cells. (B) *In vivo* PKH26-labeled cells migrated into Matrigel in the presence or absence of UC-CM 8 and 16 days following transplantation. (C) Number of PKH26-labeled fibroblasts, HUVECs and UC-MSCs in response to UC-CM was calculated as the PKH26 stained unit of each Matrigel section. Data are presented as the mean ± standard error of the mean of eight sections (n=5 mice/group). UC-CM induced significantly higher levels of migration in the HUVECs compared with DMEM treatment for 8 (^***^P<0.0001) and 16 days (^*^P<0.05). The number of UC-MSCs transplanted into the Matrigel was significantly different between the UC-CM and control group (^***^P<0.0001). Scale of bar=100 *μ*m. HUVECs, human umbilical vein endothelial cells; UC-MSCs, umbilical cord mesenchymal stem cells; UC-CM, UC-MSCs conditioned medium; DMEM, Dulbecco’s modified Eagle’s medium.

To investigate the ability of UC-CM to attract UC-MSCs, HUVECs and fibroblasts *in vivo*, the recruitment of cells into a Matrigel implant was analyzed in C57BL/6 mice. Flow cytometry and fluorescent microscopy (Fig. 2A) demonstrated that >95% of the UC-MSCs, HUVECs and fibroblasts were labeled with PKH26. The abilities of the transplanted cells to invade through the Matrigel in response to cytokines in the UC-CM were then assessed (Fig. 2B). At day 8 following implantation, the UC-MSCs were only detected in the implants containing DMEM [48±11 cells/high power field (HPF)]and UC-CM (80±14 cells/HPF). HUVECs were detected in Matrigel containing UC-CM at day 8, but not in the Matrigel containing DMEM. Starting from day 16, the number of HUVECs inside the implants increased significantly when induced by UC-CM (93±8 cells/HPF) compared with the DMEM-induced migration (61±10 cells/HPF; P<0.05). By contrast, the UC-CM did not significantly increase the invasive ability of fibroblasts compared with DMEM on days 8 or 16. The increased UC-MSC migration in response to UC-CM (127±9 cells/HPF) was significantly greater compared with that observed with DMEM (38±6 cells/HPF; P<0.001; Fig. 2C). These findings suggested that UC-CM affected the local microenvironment, which facilitated the migration of resident stem/progenitor cells in response to the chemoattractants and may reinforce tissue repair.

### Expression of CXCR4, CCR2 and c-met receptors in the UC-MSCs, HUVECs and fibroblasts

**Figure 3:**
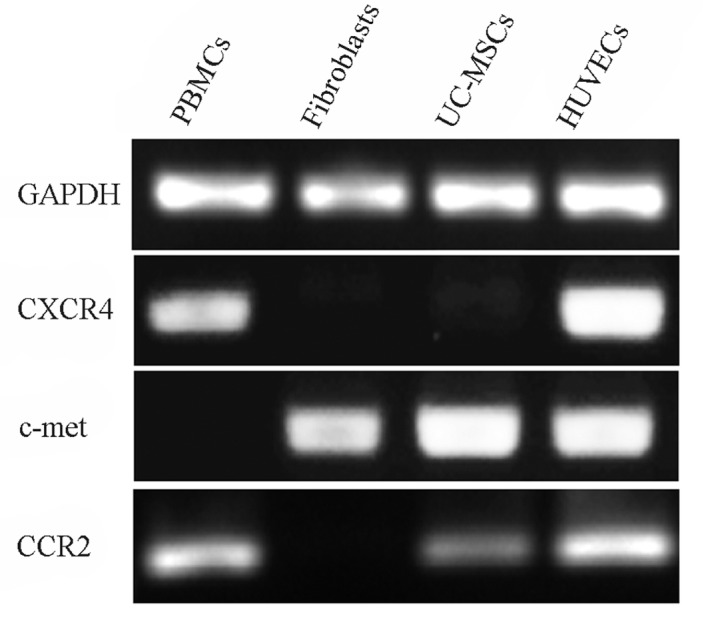
Reverse transcription-quantitative polymerase chain reaction of the expression levels of CXCR4, c-met and CCR2. Lane 1, CD3-activated PBMCs; lane 2, fibroblasts; lane 3, UC-MSCs; lane 4, HUVECs. CXCR4, C-X-C chemokine receptor 4; CCR2, C-C chemokine receptor 2; PBMCs, peripheral blood mononuclear cells; UC-MSCs, umbilical cord mesenchymal stem cells; HUVECs, human umbilical vein endothelial cells.

**Figure 4:**
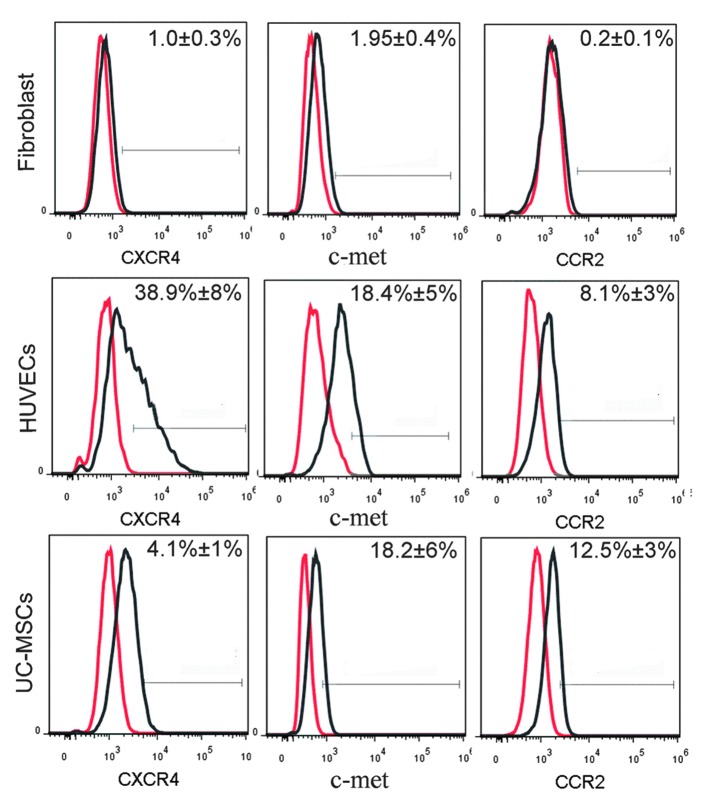
Analysis of the extracellular expression levels of CXCR4, c-met and CCR2 in fibroblasts, HUVECs and UC-MSC using flow cytometry. The cells were detached using 0.25% trypsin and analyzed using PerCP/Cy5.5-labeled anti-human CCR2, PE-labeled anti-human CXCR4 and fluorescein isothio-cyanate-labeled anti-human c-met antibodies. Red lines represent antibody isotype controls, and black lines represent the expression of the indicated markers. CXCR4, C-X-C chemokine receptor 4; CCR2, C-C chemokine receptor 2; HUVECs, human umbilical vein endothelial cells; UC-MSCs, umbilical cord mesenchymal stem cells.

The *in vivo* migration assay demonstrated that the UC-CM contributed to the recruitment of transplanted cells. To investigate the effect of the SDF-1/CXCR4, MCP-1/CCR2 and HGF/c-met axes on the migration of UC-MSCs, HUVECs and fibroblasts, the expression levels of the CXCR4, CCR2 and c-met receptors were measured (Fig. 3). The GAPDH gene was used as an internal control for the expression of mRNA. The expression of CXCR4 was significantly higher in the HUVECs compared with the UC-MSCs, and was not detected in the fibroblasts. RT-qPCR demonstrated that the expression of c-met was positive in the UC-MSCs, HUVECs and fibroblasts. By contrast, the expression of CCR2 was positive in the UC-MSCs and HUVECs, but negative in the fibroblasts. These results were confirmed using flow cytometry (Fig. 4). The data collected indicated that 38.9±8% of the HUVECs expressed CXCR4, which was 10-fold higher compared with the UC-MSCs. In addition, >18% of the UC-MSCs and HUVECs expressed c-met, although the fibroblasts expressed significantly lower levels compared with the UC-MSCs. A total of 12.5±3% of the UC-MSCs and 8.1±3% of the HUVECs expressed CCR2, however, this receptor was almost undetectable in the fibroblasts.

### UC-CM increases the migratory capacity of cells

**Figure 5:**
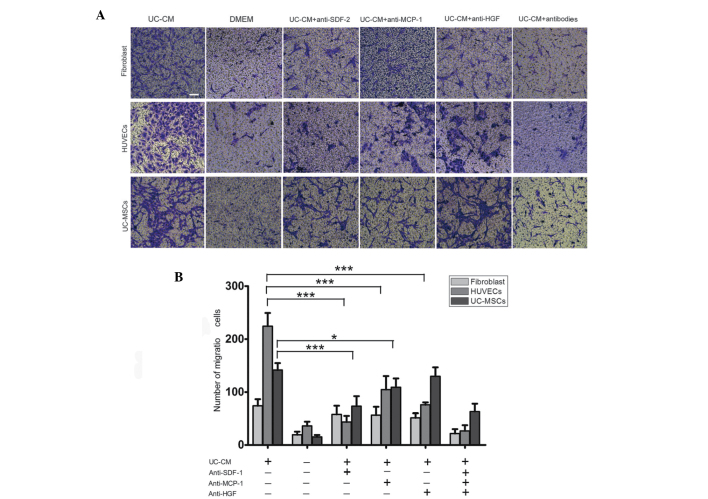
Migration of fibroblasts, HUVECs and UC-MSCs in response to UC-CM. (A) A total of 5×10^4^ cells were collected and allowed to migrate. Lane 1, UC-CM; lane 2, DMEM; lanes 3–6, in the presence or absence of anti-SDF-1 (20 *μ*g/ml), anti-MCP-1 (20 *μ*g/ml) or anti-HGF (20 *μ*g/ml), respectively. Results are from a representative experiment and are expressed as the mean number of migrated cells in three random fields, scale bar=200 *μ*m. Cells that crossed the matrigel membrane were stained with crystal violet (magnification, ×40). (B) Graphical presentation of the quantified data, presented as the number of migrated cells and expressed as the mean ± standard error of the mean. HUVECs, human umbilical vein endothelial cells; UC-MSCs, umbilical cord mesenchymal stem cells; UC-CM, UC-MSCs conditioned medium; DMEM, Dulbecco’s modified Eagle’s medium; SDF-1, stromal cell-derived factor 1; MCP-1, monocyte chemotactic protein 1; HGF, hepatocyte growth factor.

A migration assay was used to investigate the role of the cytokines in UC-CM in promoting cell migration and to determine whether the cells receptors were involved. The migration of UC-MSCs, HUVECs and fibroblasts from the upper chamber across the membrane was significantly higher in the UC-CM group compared with the DMEM group (Fig. 5A and B). As the investigation identified the expression of CXCR4, CCR2 and c-met, receptors involved in cell migration toward SDF-1, MCP-1 and HGF, on the cell surface, an antibody-based blocking assay was performed. The UC-CM significantly increased the migration of HUVECs, which was blocked by the anti-SDF-1 (P<0.001), anti-MCP-1 (P<0.001) and anti-HGF antibodies (P<0.001). The UC-CM-induced migration of UC-MSCs was almost eradicated by blocking SDF-1 with an anti-SDF-1 antibody, or MCP-1 with an anti- MCP-1 antibody. These results suggested that SDF-1, MCP-1 and HGF were involved in the UC-CM-induced migration of HUVECs via the SDF-1-CXCR4, MCP-1-CCR2 and HGF-c-met axes. Similar to the HUVECs, the SDF-1-CXCR4 and MCP-1-CCR2 axes may also be involved in the migration of UC-MSCs. By contrast, no significant alteration in migratory activity was observed in the fibroblasts in response to the neutralized antibodies.

**Figure 6:**
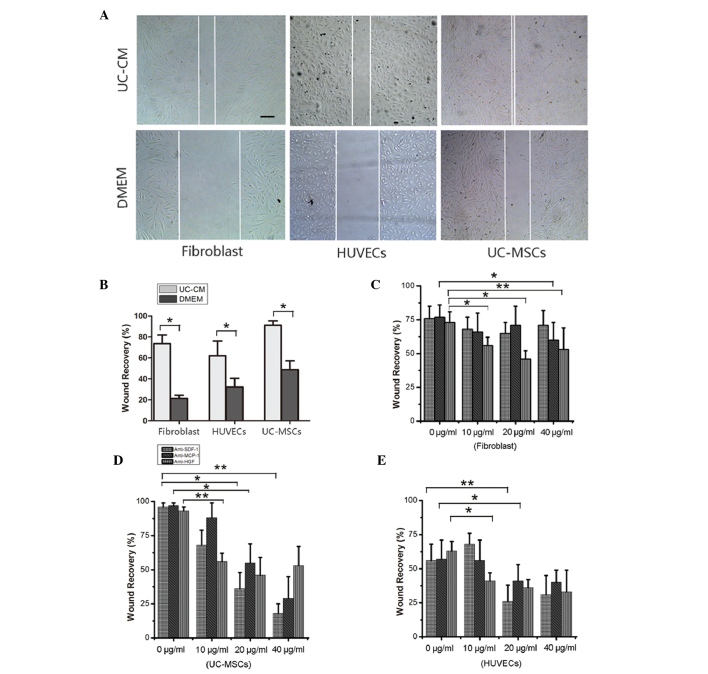
Cell migration analyzed using wound-healing assays. (A) Representative images of *in vitro* wound-healing assays in fibroblasts, HUVECs and UC-MSCs in the presence of UC-CM, vs. DMEM. Scale bar=200 *μ*m. (B) Quantification of *in vitro* wound healing, There was a significant increase in the wound closure in fibroblasts, HUVECs and UC-MSCs exposed to UC-CM compared with DMEM at 6 h (^*^P<0.05). The migration of (C) fibroblasts, (D) UC-MSCs and (E) HUVECs in response to UC-CM were inhibited by specific antibodies against known receptors. Anti-SDF-1, -MCP-1 and -HGF antibodies were added to UC-CM at concentrations of 10, 20 and 40 *μ*g/ml. A concentration-dependent reduction in cell migration was observed. ^*^P<0.05, ^**^P<0.01 and ^***^P<0.001, vs. UC-CM, determined with analysis of variance followed by Bonferroni’s post-hoc test (n=3 per group). Data are presented as the mean ± standard error of the mean. HUVECs, human umbilical vein endothelial cells; UC-MSCs, umbilical cord mesenchymal stem cells; UC-CM, UC-MSCs conditioned medium; DMEM, Dulbecco’s modified Eagle’s medium; SDF-1, stromal cell-derived factor 1; MCP-1, monocyte chemotactic protein 1; HGF, hepatocyte growth factor.

Subsequently, a wound-healing assay was performed, in which cell monolayers were scratched and cell growth and migration were quantified (Fig. 6A). The results demonstrated that incubation with UC-CM enhanced the migration of cells toward the wound, reducing its surface area (Fig. 6B). The ability of cells to migrate towards a cytokine gradient was determined using antibody blocking assays. Notably, the fibroblasts migrated in response to different concentrations of MCP-1 and HGF in a dose-dependent manner (Fig. 6C). The UC-MSCs treated with cytokine antibodies exhibited significantly reduced cell migration and wound recovery in response to the inhibition of SDF-1, MCP-1 and HGF (P<0.01). In addition, the UC-CM-induced migration of HUVECs was markedly inhibited in the presence of the anti-SDF-1, anti-HGF or MCP-1 antibodies, confirming that SDF-1, MCP-1 and HGF in the UC-CM were important for cell proliferation and/or migration.

## Discussion

The clinical application of UC-MSCs has been reported for several diseases, and the paracrine effects of UC-MSCs may contribute to these beneficial effects (24,25). In the present study, *in vitro* experiments demonstrated that UC-CM supported tube formation and stimulated the migration of UC-MSCs and HUVECs. Therefore, CM harvested from UC-MSCs may enhance the positive effects of cellular-based therapy. However, the factors and mechanisms responsible for stimulating the migration of cells towards wounded microenvironments, remain to be fully elucidated. Tissue repair is a complex process, which requires the collaboration of various factors and cells (26). It is likely that UC-CM contains high levels of growth factors and chemokines, which may contribute to a chemoattractive environment to circulating progenitor and stem cells in adjacent tissues (27). A previous study demonstrated that MSCs are likely to possess chemotactic properties in injury tissue (28), and HUVECs are involved in blood vessel remodeling (29). Fibroblasts however, contribute to the maintenance and regeneration of connective tissues (30). In the present study, the expression levels of specific cell surface receptors were assessed in UC-MSCs, HUVECs and fibroblasts, and the cells were labeled with PKH26 for *in vivo* cell tracking and chemoinvasion assays.

**Figure 7:**
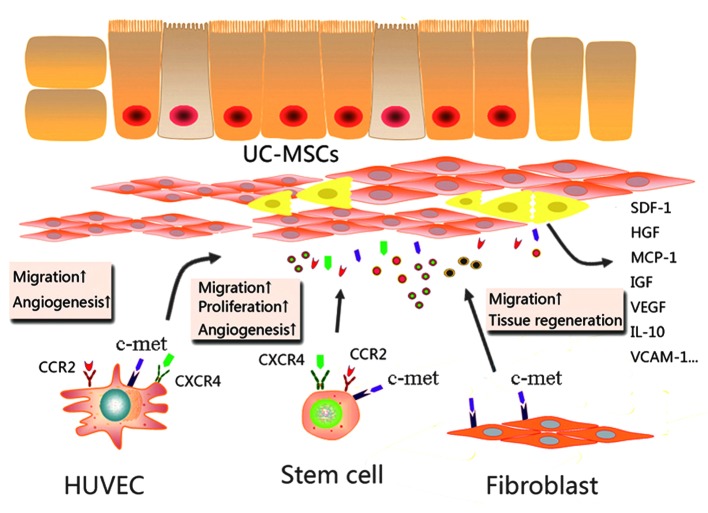
A model of the paracrine mechanisms of UC-MSCs in tissue repair. In damaged tissues, UC-MSCs attract stem/progenitor cells via paracrine activity involving SDF-1/CXCR4 and MCP-1/CCR2 interaction. Potent paracrine chemoattractant and angiogenic factors affect the microenvironment by acting on different cell types, leading to tissue repair and angiogenesis. UC-MSCs, umbilical cord mesenchymal stem cells; SDF-1, stromal cell-derived factor 1; CXCR4, C-X-C chemokine receptor 4; c-met, MCP-1, monocyte chemotactic protein 1; HGF, hepatocyte growth factor; CCR2, C-C chemokine receptor 2; HUVECs, human umbilical vein endothelial cells; IGF, insulin-like growth factor; VEGF, vascular endothelial growth factor; IL, interleukin; VCAM, vascular cell adhesion protein.

Previous studies have reported that UC-MSCs secrete certain cytokines and factors (14,31–33), similar to other stem cells. However, the relative expression levels of these factors and the importance of UC-MSC-derived cytokines in tissue repair remain to be elucidated. In the present study, seven factors, known for their angiogenic and chemotactic properties, were investigated (Table I). The data revealed that UC-MSCs secreted significantly increased the levels of IGF-1 (871±80 pg/ml), IL-8 (1,444±225 pg/ml) and HGF (643±31 pg/ml), however, markedly lower levels of the two angiogenic factors, PDGF-BB (38.5±9 pg/ml) and FGF-2 (59±13 pg/ml) were observed. This suggested that IGF-1, IL-8 and HGF, rather than PDGF-BB and FGF-2, may be responsible for the angiogenic potential of UC-CM. Notably, the UC-MSCs produced higher levels of BDNF (13,900±2156 pg/ml), which can enhance the growth, differentiation and survival of neurons (34) This suggested additional potential applications for UC-CM. SDF-1, MCP-1 and HGF can be isolated from the UC-MSCs in large quantities compared with other chemotactic factors in UC-CM. Several studies have demonstrated that SDF-1, MCP-1 and HGF are able to induce the homing and migration of various types of cells (14,35). Therefore, it was hypothesized that SDF-1, MCP-1 and HGF may be key regulators in UC-CM, and that growth factors and chemokines secreted by the UC-MSCs injected into an injured area, attract circulating progenitor/stem cells, which migrate and infiltratd into the tissue and initiate regeneration (Fig. 7). The present study demonstrated that SDF-1, MCP-1 and HGF were secreted by the UC-MSCs and were able to mediate productive repair by recruiting reparative cells with specific cell receptors. Specifically, UC-MSCs and HUVECs were able to migrate *in vitro* and *in vivo*, in response to chemotactic factors secreted by the UC-MSCs.

Based on the expression levels of CXCR4, the role of the SDF-1-CXCR4 axis in chemotactic actions of UC-CM was analyzed using a Matrigel migration assay. The UC-CM was incubated with neutralizing antibodies against SDF-1, which suppressed the chemotactic response of the HUVECs (P<0.001) and UC-MSCs (P<0.01) to UC-CM (Fig. 5). When the antibodies were added to inhibit the effects of the SDF-1-CXCR4 axis in the wound-healing assay, the data revealed that HUVECs exhibited a greater migratory ability in the presence of 20 *μ*g/ml anti-SDF-1 (P<0.01) compared with the UC-MSCs (P<0.05;Fig. 6), suggesting that the SDF-1 in the UC-CM was responsible for chemotaxis. Consistent with these results, the UC-MSCs and HUVECs expressed detectable levels of CXCR4, as determined by flow cytometry and RT-qPCR, wheras CXCR4 was not detected in the fibroblasts. SDF-1 stimulates the recruitment of progenitor cells to ischemic tissue (32,36). The present study demonstrated that SDF-1 not only induced the concentration-dependent migration of UC-MSCs and HUVECs, but promoted cell proliferation.

MCP-1 is also important in cell migration. Previous studies identified the expression of the MCP-1 receptor, CCR2, in BM-MSCs, however, reports describing MSC migration in response to MCP-1 are conflicting (33,37). In the present study, assays were performed in the presence of an MCP-1 neutralizing antibody. As expected, the numbers of migrated UC-MSCs (P<0.05) and HUVECs (P<0.001) were significantly reduced in the presence of the antibody. The wound-healing assay confirmed these findings; the repair of scratch wounds in the UC-MSCs and HUVECs was significantly slower following treatment with 20 *μ*g/ml anti-MCP-1. Therefore, it is likely that the signal transduction pathways involved in MCP-1/CCR2-mediated cell migration are cell type-specific, and that the expression of CCR2 on the cell surface was critical in this process.

HGF is a pleiotropic cytokine, which promotes epithelial and endothelial cell proliferation and invasion through the extracellular matrix (38,39). The HGF-c-met axis is important in enhancing the engraftment of MSCs in the injured heart (40). To further investigate whether the cell migration was mediated by HGF-c-met signaling, Matrigel migration and scratch wound healing assays were performed. The chemotactic effects of UC-CM treated with anti-HGF on the HUVECs were significantly inhibited compared with the control (P<0.001). By contrast, the migration of UC-MSCs and fibroblasts were equivalent to those of the control (Fig. 5). Notably, the extracellular expression of c-met was detected on the UC-MSCs and HUVECs, but not on the fibroblasts (Figs. 3 and 4), suggesting that the HGF-c-met axis was only responsible for the migration of HUVECs. In addition, the scratch wound healing assay indicated that wound closure in the UC-MSCs, HUVECs and fibroblasts was significantly slower in the presence of anti-HGF antibodies. It was hypothesized that HGF enhanced the rate of wound closure by promoting cell proliferation (41,42).

The present study hypothesized that at least two factors within the UC-CM induced chemotaxis. UC-CM may also attract and enhance the proliferation of target cells and induce tube formation. The antibody blocking experiment resulted in significantly reduced cell migration compared with single antibody experiments (Figs. 5 and 6). Therefore, the present study concluded that UC-CM induced the migratory activity of cells via the SDF-1/CXCR4 axis and via the binding of MCP-1 to CCR2. It is likely that there are multiple complex paracrine factors within UC-CM, rather than one single molecule (Fig. 7).

Taken together, the data presented in the present study revealed a mechanism, whereby UC-CM exerted significant angiogenic abilities and chemoattractant effects on progenitor cells, fibroblasts and stem cells. These results suggest a role for the SDF-1/CXCR4 and MCP-1/CCR2 axes in UC-CM-induced migration. The local delivery of UC-CM may induce the recruitment of cells from the surrounding tissues and enhance the proliferation of these cells in injured tissue. Therefore, the use of UC-CM may be suitable for regenerative medicine.

## Figures and Tables

**Table I:** Cytokines and growth factor levels present in conditioned medium derived from UC-MSCs and fibroblasts.

Cytokine	Assay	Conditioned medium (pg/ml)	
		UC-MSC (n=3)	Fibroblast (n=3)
BDNF	ELISA	13,900.25±2156.17	ND
SDF-1	ELISA	770.63±45.36^a^	74.44±8.23
IGF	ELISA	871.28±80.29^a^	27±11.43
VCAM-1	ELISA	549.44±63.32	N/A
TGF-β	ELISA	4,330.36±798.19^a^	1,605.86±335.36
HGF	ELISA	643.05±31.91	N/A
VEGF	LC	224.06±47.42	340.75±117.09^c^
EGF	LC	<5.40±0.00	<3.60±0.00
FGF-2	LC	59.55±13.64^b^	28.90±9.15
PDGF-BB	LC	38.05±9.05^b^	29.10±12.21
IL-10	LC	<4.00±0.00	1.66±0.60
IL-8	LC	1,444.60±225.33^a^	285.61±172.00
IP-10	LC	34.80±6.19	36.40±15.17
TGF-α	LC	<0.4±0.00	ND
MCP-1	LC	13,038.81±1134.06^a^	914.23±213.06
SCF	LC	<1.25±0.00	ND

Data are expressed as the mean ± standard deviation.

a P<0.001 and

b P<0.01, compared with the fibroblast group;

c P<0.01, compared with the UC-MSC group. LC, liquidchip assay; ND, not detectable; N/A, not available; BDNF, brain-derived neurotrophic factor; SDF, stromal cell-derived factor; IGF, insulin-like growth factor; VCAM, vascular cell adhesion molecule; TGF, transforming growth factor; HGF, hepa-tocyte growth factor; VEGF, vascular endothelial growth factor; EGF, epidermal growth factor; FGF, fibroblast growth factor; PDGF-BB, platelet-derived growth factor-BB; IL, interleukin; IP, interferon-inducible protein; MCP, monocyte chemotactic protein; SCF, stem cell factor.

## References

[1] Wang HS, Hung SC, Peng ST (2004). Mesenchymal stem cells in the Wharton’s jelly of the human umbilical cord. Stem Cells.

[2] Madlambayan G, Rogers I (2006). Umbilical cord-derived stem cells for tissue therapy: current and future uses. Regen Med.

[3] Ruan ZB, Zhu L, Yin YG, Chen GC (2010). The mechanism underlying the differentiation of human umbilical cord-derived mesenchymal stem cells into myocardial cells induced by 5-azacytidine. Indian J Med Sci.

[4] Schneider RK, Püllen A, Kramann R (2010). Long-term survival and characterisation of human umbilical cord-derived mesenchymal stem cells on dermal equivalents. Differentiation.

[5] Rabb H (2005). Paracrine and differentiation mechanisms underlying stem cell therapy for the damaged kidney. Am J Physiol Renal Physiol.

[6] Deschepper M, Oudina K, David B (2011). Survival and function of mesenchymal stem cells (MSCs) depend on glucose to overcome exposure to long-term, severe and continuous hypoxia. J Cell Mol Med.

[7] Nagaya N, Kangawa K, Itoh T (2005). Transplantation of mesenchymal stem cells improves cardiac function in a rat model of dilated cardiomyopathy. Circulation.

[8] Müller-Ehmsen J, Krausgrill B, Burst V (2006). Effective engraftment but poor mid-term persistence of mononuclear and mesenchymal bone marrow cells in acute and chronic rat myocardial infarction. J Mol Cell Cardiol.

[9] Zhao JJ, Liu JL, Liu L, Jia HY (2014). Protection of mesenchymal stem cells on acute kidney injury. Mol Med Rep.

[10] Chen Y, Xiang LX, Shao JZ (2010). Recruitment of endogenous bone marrow mesenchymal stem cells towards injured liver. J Cell Mol Med.

[11] Tasso R, Augello A, Boccardo S (2009). Recruitment of a host’s osteoprogenitor cells using exogenous mesenchymal stem cells seeded on porous ceramic. Tissue Eng Part A.

[12] Tögel F, Isaac J, Hu Z, Weiss K, Westenfelder C (2005). Renal SDF-1 signals mobilization and homing of CXCR4-positive cells to the kidney after ischemic injury. Kidney Int.

[13] Ma J, Ge J, Zhang S (2005). Time course of myocardial stromal cell-derived factor 1 expression and beneficial effects of intravenously administered bone marrow stem cells in rats with experimental myocardial infarction. Basic Res Cardiol.

[14] Son BR, Marquez-Curtis LA, Kucia M (2006). Migration of bone marrow and cord blood mesenchymal stem cells in vitro is regulated by stromal-derived factor-1-CXCR4 and hepatocyte growth factor-c-met axes and involves matrix metalloproteinases. Stem Cells.

[15] Somerset DA, Li XF, Afford S (1998). Ontogeny of hepatocyte growth factor (HGF) and its receptor (c-met) in human placenta: reduced HGF expression in intrauterine growth restriction. Am J Pathol.

[16] Liao WT, Yu HS, Arbiser JL (2010). Enhanced MCP-1 release by keloid CD14+ cells augments fibroblast proliferation: role of MCP-1 and Akt pathway in keloids. Exp Dermatol.

[17] Furuichi K, Wada T, Iwata Y (2003). CCR2 signaling contributes to ischemia-reperfusion injury in kidney. J Am Soc Nephrol.

[18] Shyu WC, Lee YJ, Liu DD, Lin SZ, Li H (2006). Homing genes, cell therapy and stroke. Front Biosci.

[19] Ashrafpour H, Huang N, Neligan PC (2004). Vasodilator effect and mechanism of action of vascular endothelial growth factor in skin vasculature. Am J Physiol Heart Circ Physiol.

[20] Morimoto N, Saso Y, Tomihata K (2005). Viability and function of autologous and allogeneic fibroblasts seeded in dermal substitutes after implantation. J Surg Res.

[21] Lamme EN, van Leeuwen RT, Mekkes JR, Middelkoop E (2002). Allogeneic fibroblasts in dermal substitutes induce inflammation and scar formation. Wound Repair Regen.

[22] Cavallari G, Olivi E, Bianchi F (2012). Mesenchymal stem cells and islet cotransplantation in diabetic rats: improved islet graft revas-cularization and function by human adipose tissue-derived stem cells preconditioned with natural molecules. Cell Transplant.

[23] Wynn RF, Hart CA, Corradi-Perini C (2004). A small proportion of mesenchymal stem cells strongly expresses functionally active CXCR4 receptor capable of promoting migration to bone marrow. Blood.

[24] Dai W, Hale SL, Kloner RA (2007). Role of a paracrine action of mesen-chymal stem cells in the improvement of left ventricular function after coronary artery occlusion in rats. Regen Med.

[25] Li Z, Guo J, Chang Q, Zhang A (2009). Paracrine role for mesen-chymal stem cells in acute myocardial infarction. Biol Pharm Bull.

[26] Ohnishi S, Sumiyoshi H, Kitamura S, Nagaya N (2007). Mesenchymal stem cells attenuate cardiac fibroblast proliferation and collagen synthesis through paracrine actions. FEBS Lett.

[27] Nakanishi C, Yamagishi M, Yamahara K (2008). Activation of cardiac progenitor cells through paracrine effects of mesenchymal stem cells. Biochem Biophys Res Commun.

[28] Dwyer RM, Potter-Beirne SM, Harrington KA (2007). Monocyte chemotactic protein-1 secreted by primary breast tumors stimulates migration of mesenchymal stem cells. Clin Cancer Res.

[29] Chen Z, Htay A, Dos Santos W (2009). In vitro angiogenesis by human umbilical vein endothelial cells (HUVEC) induced by three-dimensional co-culture with glioblastoma cells. J Neurooncol.

[30] Jacob M, Chang L, Puré E (2012). Fibroblast activation protein in remodeling tissues. Curr Mol Med.

[31] Mbalaviele G, Orcel P, Bouizar Z, Jullienne A, De Vernejoul MC (1992). Transforming growth factor-beta enhances calcitonin-induced cyclic AMP production and the number of calcitonin receptors in long-term cultures of human umbilical cord blood monocytes in the presence of 1,25-dihydroxychole-calciferol. J Cell Physiol.

[32] Peled A, Kollet O, Ponomaryov T (2000). The chemokine SDF-1 activates the integrins LFA-1, VLA-4, and VLA-5 on immature human CD34(+) cells: role in transendothelial/stromal migration and engraftment of NOD/SCID mice. Blood.

[33] Wang L, Li Y, Chen J (2002). Ischemic cerebral tissue and MCP-1 enhance rat bone marrow stromal cell migration in interface culture. Exp Hematol.

[34] Patel AV, Krimm RF (2010). BDNF is required for the survival of differentiated geniculate ganglion neurons. Dev Biol.

[35] Sohni A, Verfaillie CM (2013). Mesenchymal stem cells migration homing and tracking. Stem Cells Int.

[36] Lapidot T, Kollet O (2002). The essential roles of the chemokine SDF-1 and its receptor CXCR4 in human stem cell homing and repopulation of transplanted immune-deficient NOD/SCID and NOD/SCID/B2m(null) mice. Leukemia.

[37] Ringe J, Strassburg S, Neumann K (2007). Towards in situ tissue repair: human mesenchymal stem cells express chemokine receptors CXCR1, CXCR2 and CCR2, and migrate upon stimulation with CXCL8 but not CCL2. J Cell Biochem.

[38] Trusolino L, Comoglio PM (2002). Scatter-factor and semaphorin receptors: cell signalling for invasive growth. Nat Rev Cancer.

[39] Birchmeier C, Birchmeier W, Gherardi E, Vande Woude GF (2003). Met, metastasis, motility and more. Nat Rev Mol Cell Biol.

[40] Duan HF, Wu CT, Wu DL (2003). Treatment of myocardial ischemia with bone marrow-derived mesenchymal stem cells overexpressing hepatocyte growth factor. Mol Ther.

[41] Oyagi S, Hirose M, Kojima M (2006). Therapeutic effect of transplanting HGF-treated bone marrow mesenchymal cells into CCl4-injured rats. J Hepatol.

[42] Burgazli KM, Bui KL, Mericliler M, Albayrak AT, Parahuleva M, Erdogan A (2013). The effects of different types of statins on proliferation and migration of HGF-induced Human Umbilical Vein Endothelial Cells (HUVECs). Eur Rev Med Pharmacol Sci.

